# Supplementary figures and images for: Neuronal synchrony and the relation between the blood-oxygen-level dependent response and the local field potential
Source: PLoS Biol. 2017 Jul 24;15(7):e2001461. doi: 10.1371/journal.pbio.2001461 (PMC5524566; doi:10.1371/journal.pbio.2001461)

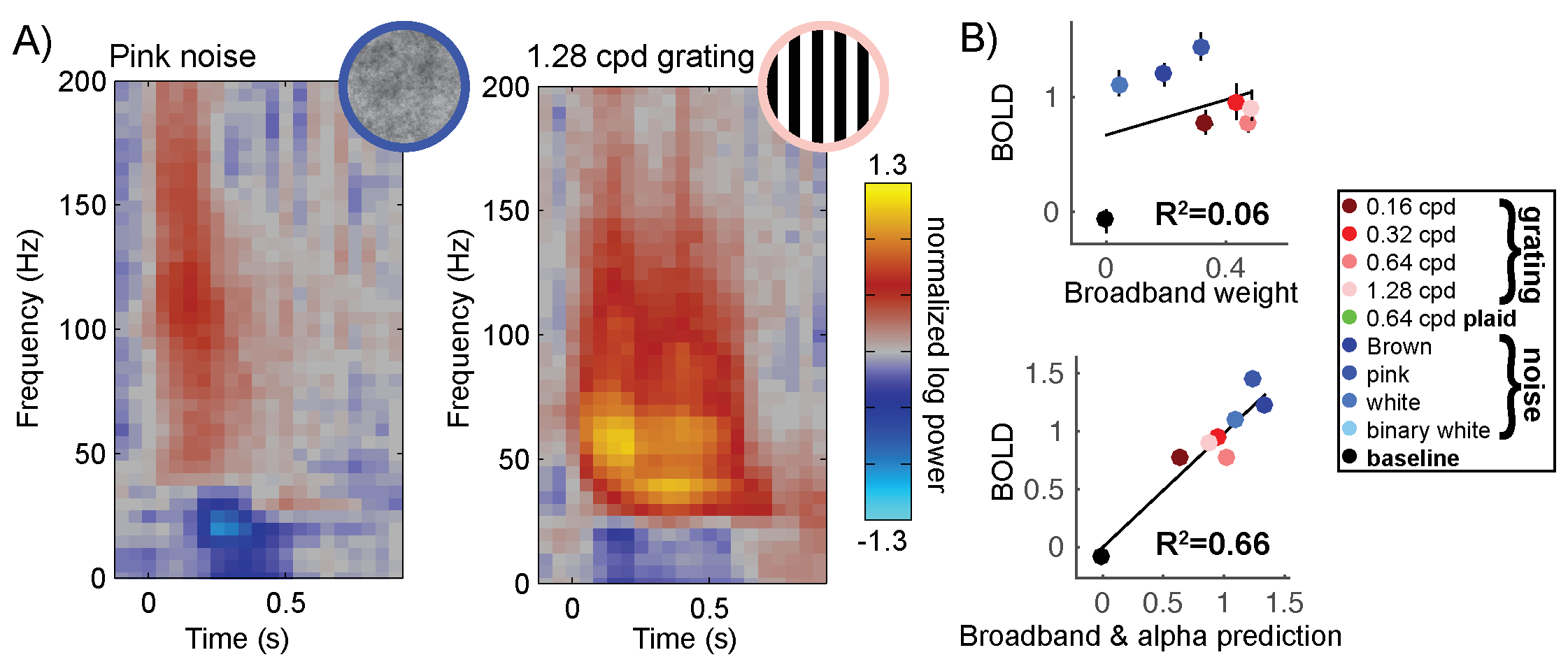

Supplement: S1 Fig — A) Time/frequency spectrograms for the pink noise pattern and the grating in an exemplary V2 electrode show that power in the low frequencies decreased more for the pink noise pattern (left) than for the grating (right). B) Top: the correlation between broadband and BOLD shows that the broadband response underpredicts the BOLD response for the noise patterns (blue dots). Red and pink dots represent the gratings. This pattern is visible in most V2/V3 electrodes (S5 Fig) Bottom: taking into account the alpha decreases in the regression model explains the variance in the BOLD response that was not explained by the broadband changes. The R2 represents the cross-validated coefficient of determination. (TIF) [file pbio.2001461.s001.tif]

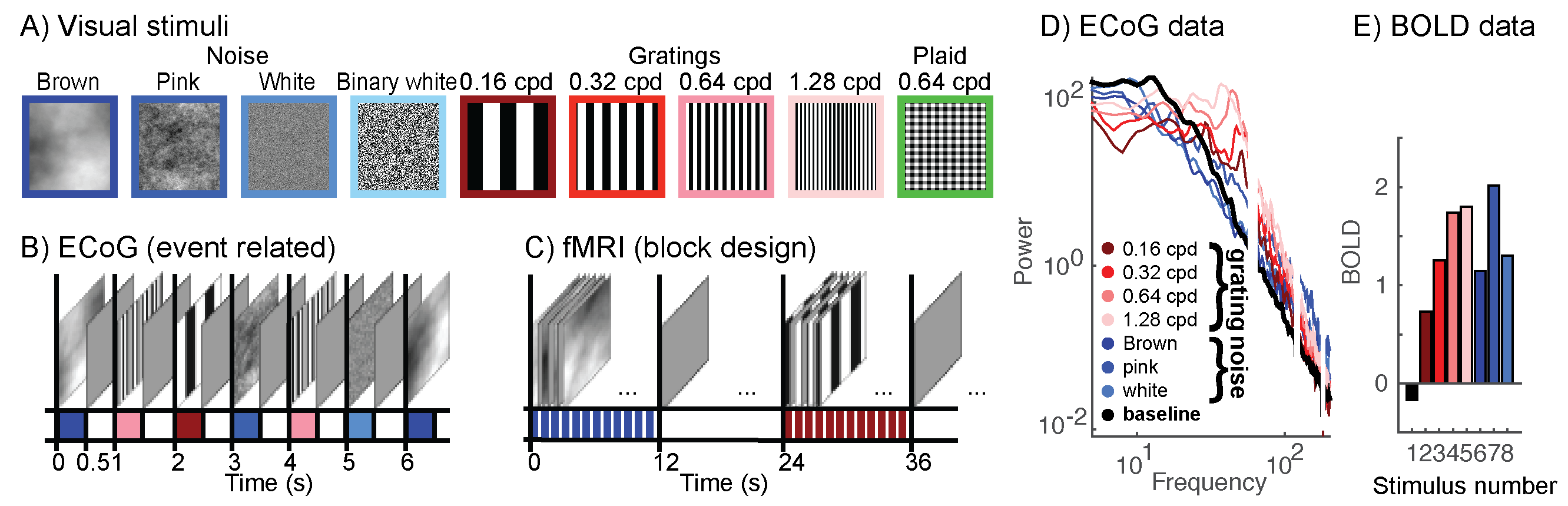

Supplement: S2 Fig — A) ECoG and fMRI responses were measured to 8 different stationary stimuli. In all experiments, subjects were instructed to fixate on a dot at the center of the screen that alternated between red and green, changing colors at random times. Subjects pressed a button when the fixation dot changed color. ECoG Subject 2 did not make manual responses because these responses were found to interfere with visual fixation. B) ECoG responses were measured in an event-related design, in which stimuli were presented every 1000 milliseconds. Stimuli were presented for 500 milliseconds followed by a blank screen. C) Stimuli were presented in blocks of 12 seconds during fMRI, followed by 12 seconds of blank. D) Example ECoG power spectrum for one electrode. ECoG data showed broadband increases (>100 Hz) compared to baseline, narrowband gamma increases around 40 HZ, and a decrease in alpha power around 10 Hz. E) The blood-oxygen-level dependent (BOLD) response increased in different levels for the different stimuli averaged across subjects. When averaging the BOLD signal across subjects, the percent signal change per subject was vector-length normalized, (BOLDnormalizedi=BOLDchangeiNORM, for condition i, in which NORM=∑iBOLDchangei2). To then reestimate the percent signal change across subjects, the averaged vector-length–normalized values were multiplied by the average norm. (TIF) [file pbio.2001461.s002.tif]

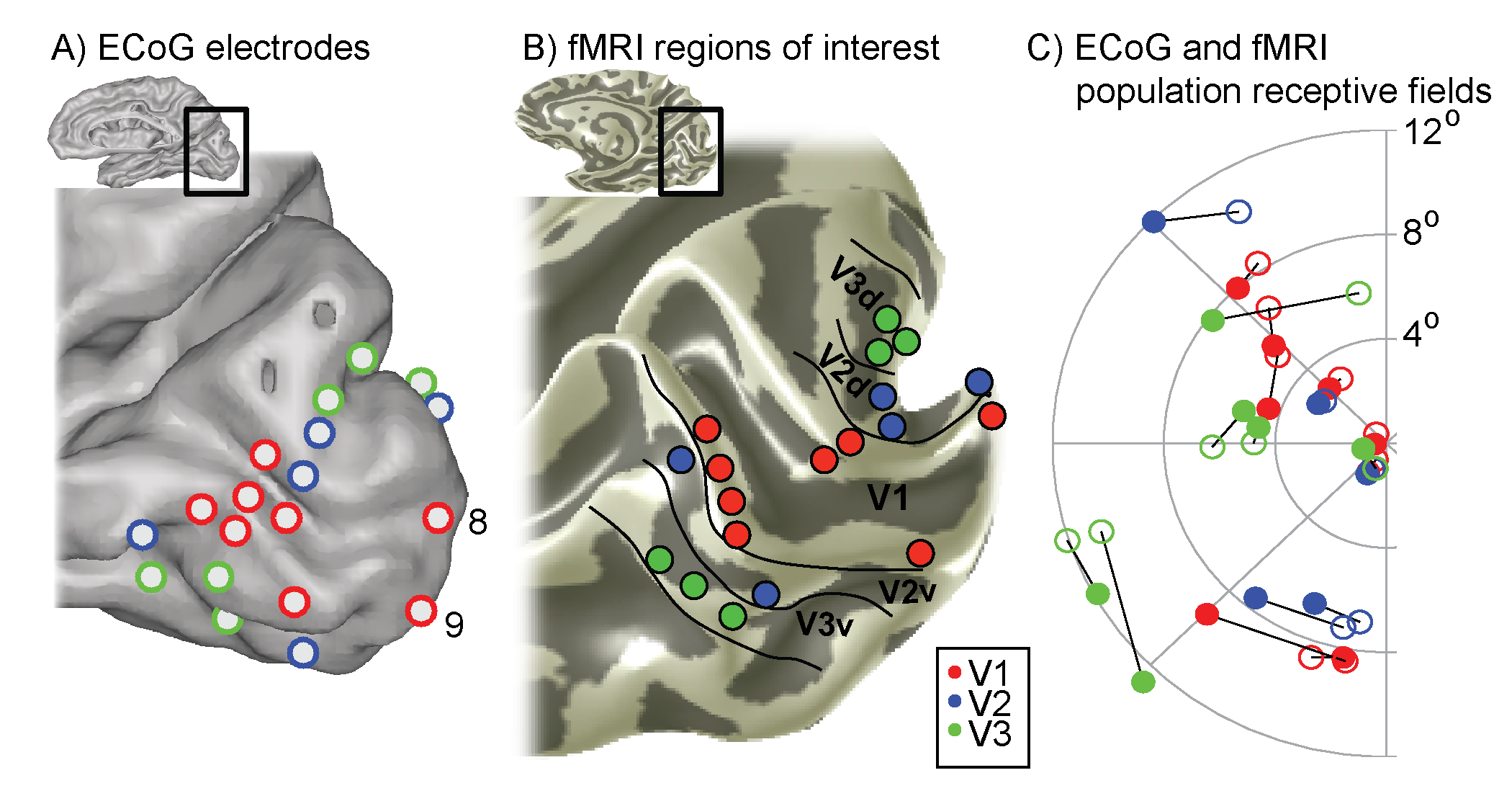

Supplement: S3 Fig — A) Channels in electrocorticographic (ECoG) S1 selected for further analysis. These channels were located within V1 (red), V2 (blue), or V3 (green), had significant broadband or gamma response to any stimuli, and had population Receptive Fields (pRF) variance explained >0.15. V1 sites 8 and 9 are indicated, since these had the largest gamma responses. B) Electrode ROIs in functional magnetic resonance imaging (fMRI) S1. Disc ROIs (radius = 2 mm) were defined to have similar anatomical and retinotopic position as the ECoG Channels. C) The pRF centers for fMRI ROIs (filled circles) were chosen to be close to those for ECoG electrodes (open circles). Because the pRF centers measured with fMRI do not completely cover the visual field map, the locations can differ slightly. (TIF) [file pbio.2001461.s003.tif]

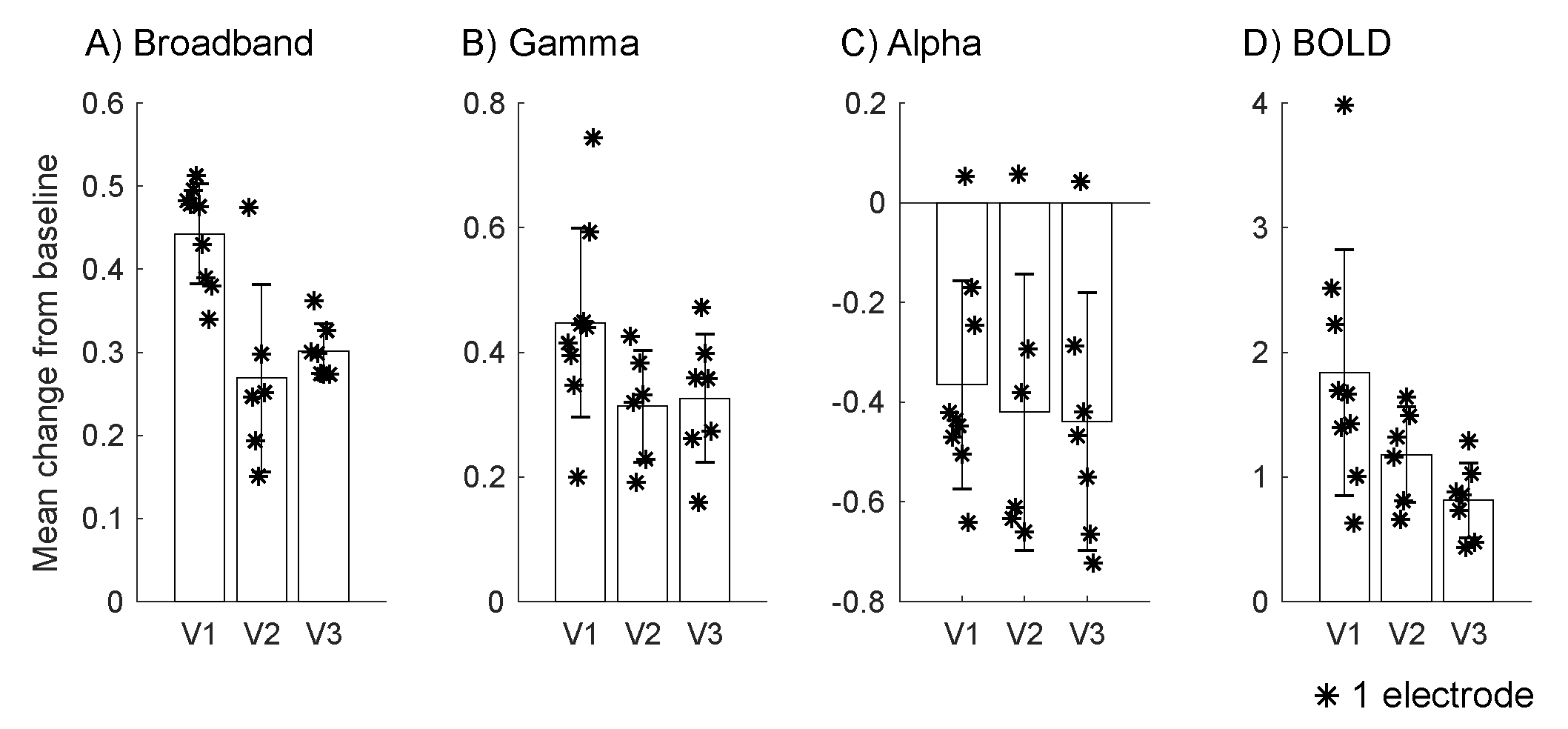

Supplement: S4 Fig — (A) For each electrocorticographic (ECoG) electrode, for each stimulus condition, the broadband change was calculated. The average log10 power from the inter stimulus baseline period was subtracted. The mean change from the baseline was then averaged across the 8–10 stimuli. (B and C) The same as A) shown for gamma and alpha. (D) For each ECoG electrode, for each stimulus condition, the blood-oxygen-level dependent (BOLD) percent signal change was calculated. The mean change from the baseline was then averaged across the stimuli. (TIF) [file pbio.2001461.s004.tif]

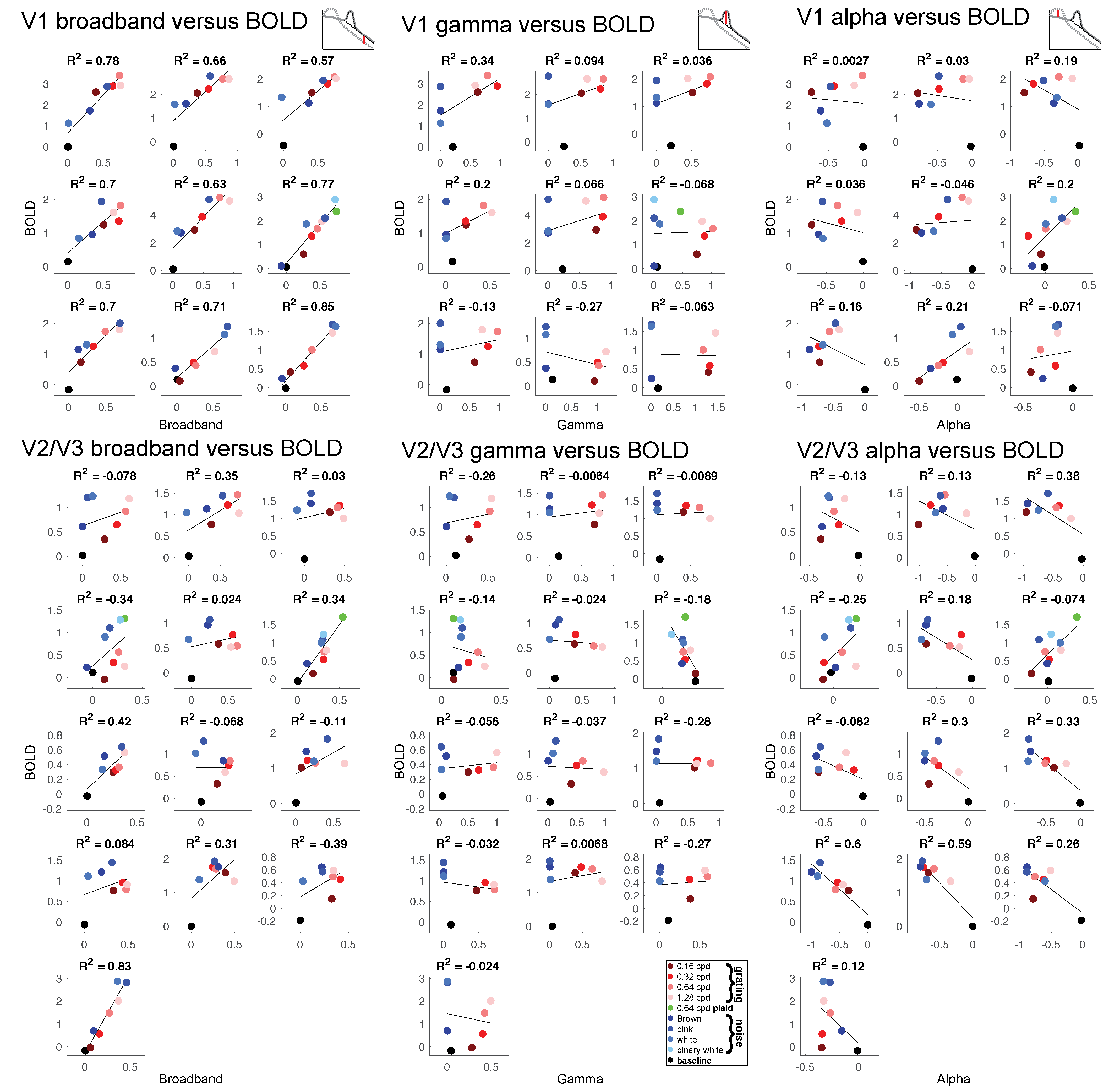

Supplement: S5 Fig — Correlation between BOLD and ECoG in V1 and V2/V3. The R2 is cross-validated: beta values are calculated from half the ECoG trials and half the functional magnetic resonance imaging (fMRI) subjects, and the regression model is tested on the other half of the trials and subjects. (TIF) [file pbio.2001461.s005.tif]

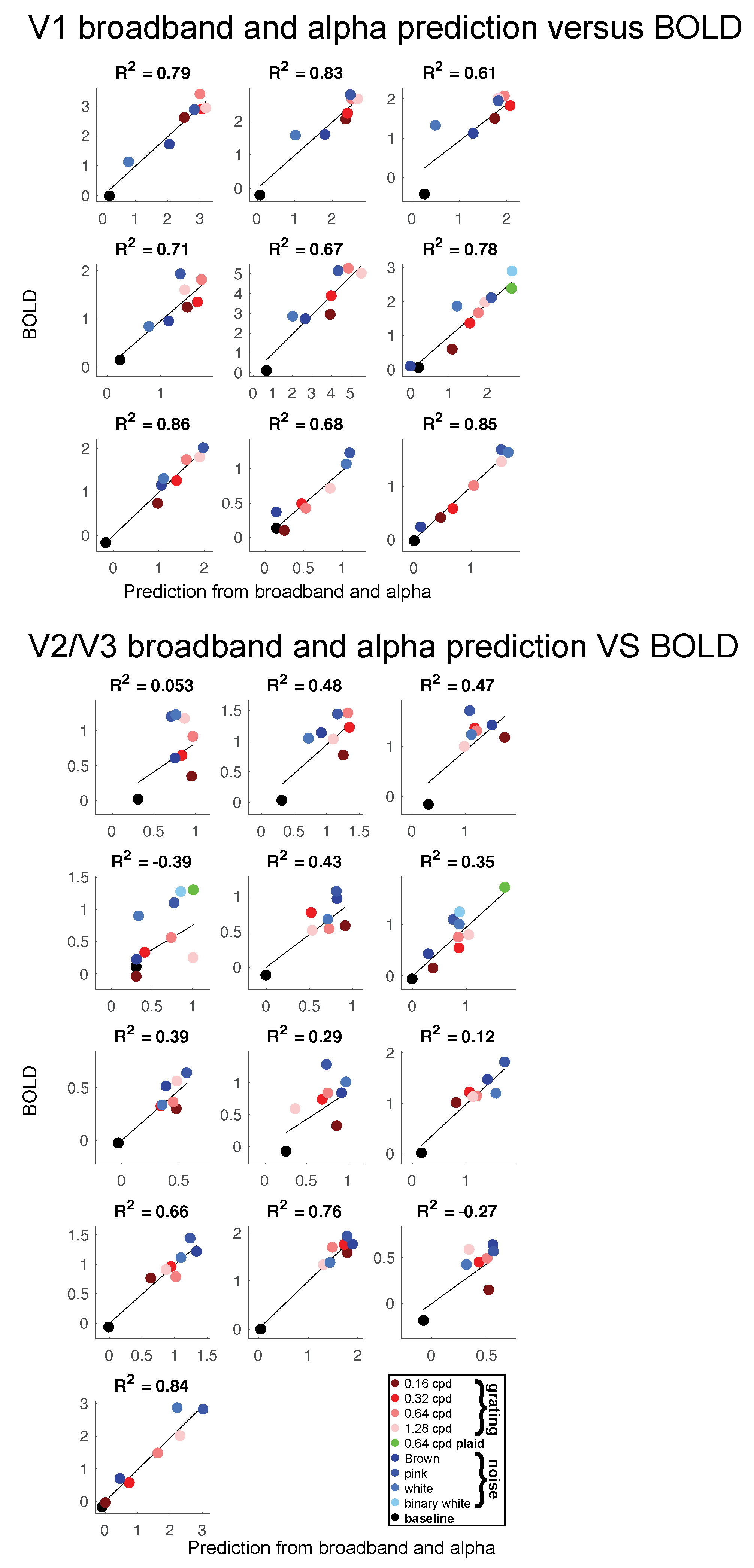

Supplement: S6 Fig — This plot shows the predicted BOLD (x-axis) versus measured BOLD (y-axis) for the 9 V1 sites (top) and 13 V2/V3 sites (bottom), based on a linear regression of the broadband and alpha components of the ECoG signals. The coefficient of determination, R2, was cross-validated. (TIF) [file pbio.2001461.s006.tif]

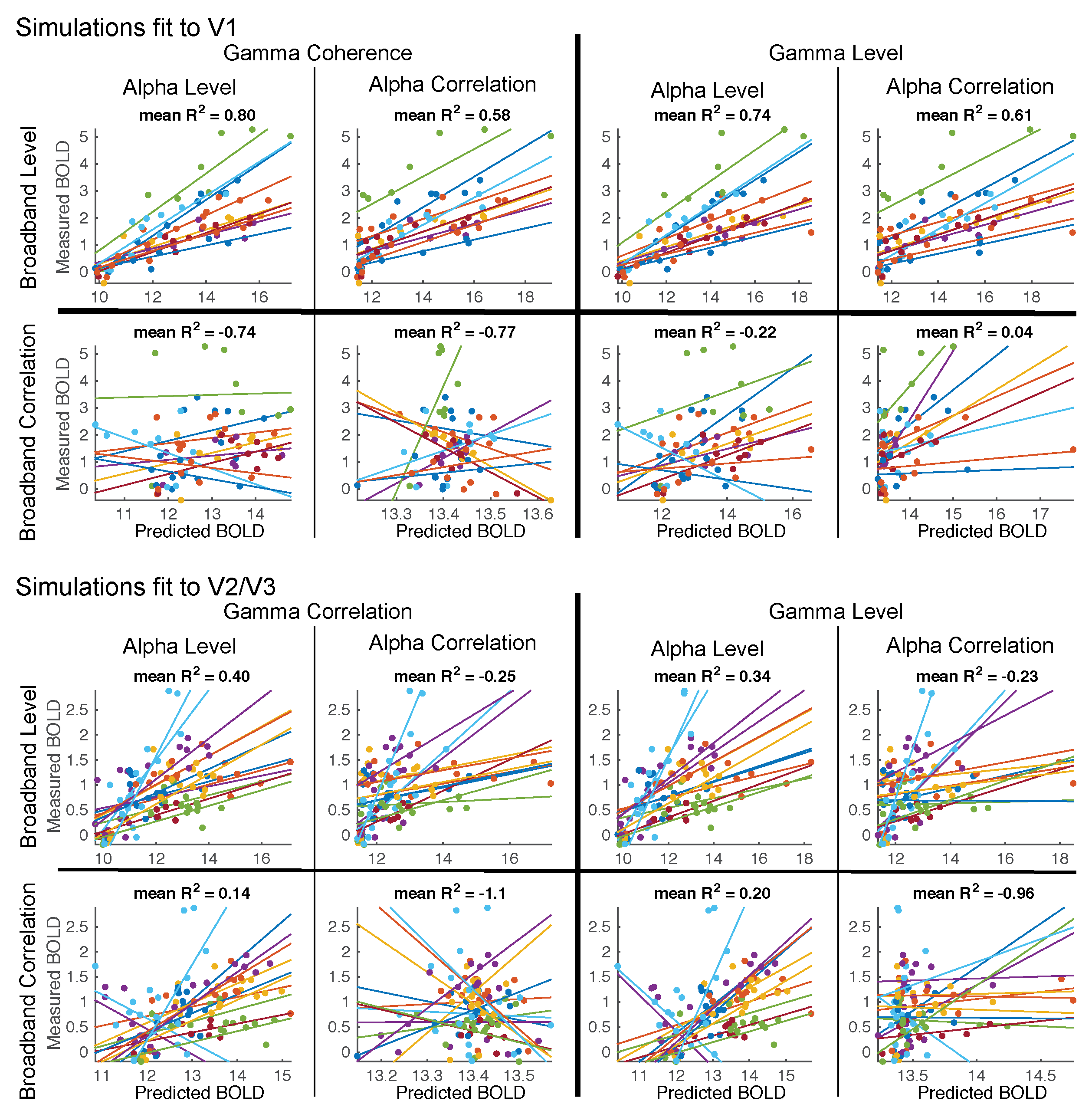

Supplement: S7 Fig — This plot shows the predicted BOLD (x-axis) versus measured BOLD (y-axis) for the 9 V1 sites (top) and 13 V2/V3 sites (bottom). Each color corresponds to one site. The cross-validated coefficient of determination (R2) was computed separately for each of the 9 sites and then averaged. The different subplots are models solved with different constraints. In the main text of the paper, model parameters were fit with three constraints: (1) the C1 (broadband) time series had a fixed, nonzero level (but could vary in correlation between neurons), (2) the C2 (gamma) time series had a fixed, nonzero level (but could vary in correlation), and (3) the C3 time series had a fixed, nonzero correlation (but could vary in level). The model predictions based on these constrains are plotted in the upper left of both the upper panel (V1) and the lower panel (V2/V3). Seven alternative models were run, and their predictions are shown in the remaining panels. For these models, the three input types, C1, C2, and C3, were constrained to have time series varying in either the level or correlations across neurons but not both. (TIF) [file pbio.2001461.s007.tif]

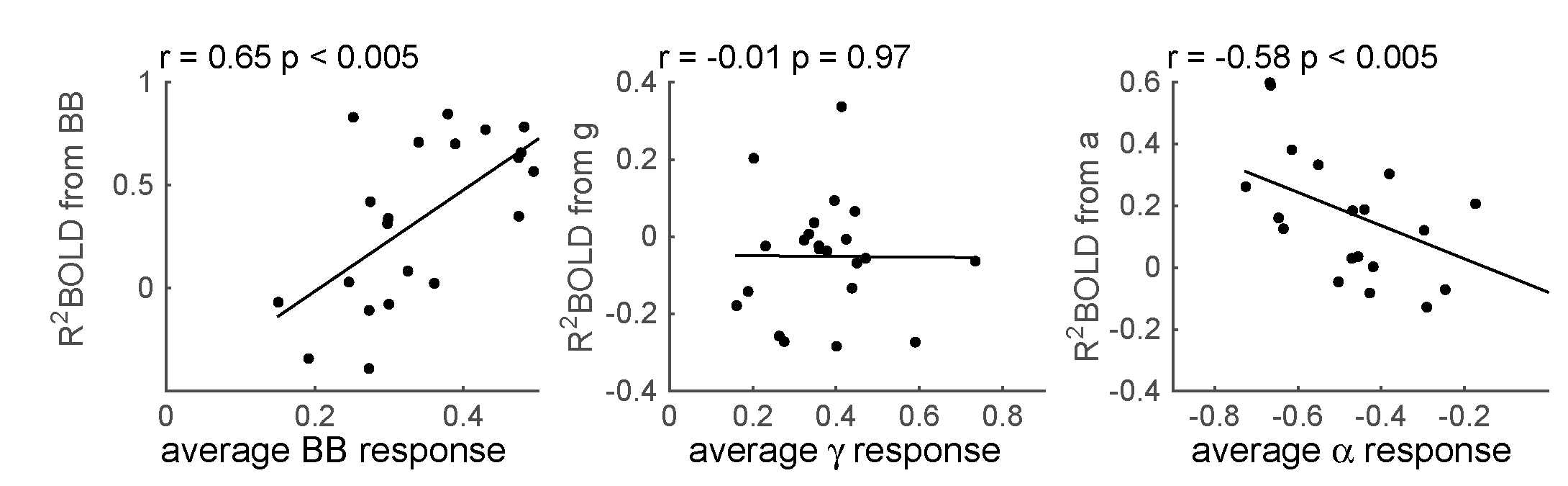

Supplement: S8 Fig — Variance in the BOLD response explained by ECoG (R2, the coefficient of determination) as a function of the size of the ECoG response. Each dot represents 1 electrode. x-axis: for each electrode, ECoG broadband, gamma, and alpha responses were averaged across (nonbaseline) stimuli. y-axis: the cross-validated R2 when BOLD is explained by broadband (left), gamma (middle), and alpha (right). (TIF) [file pbio.2001461.s008.tif]

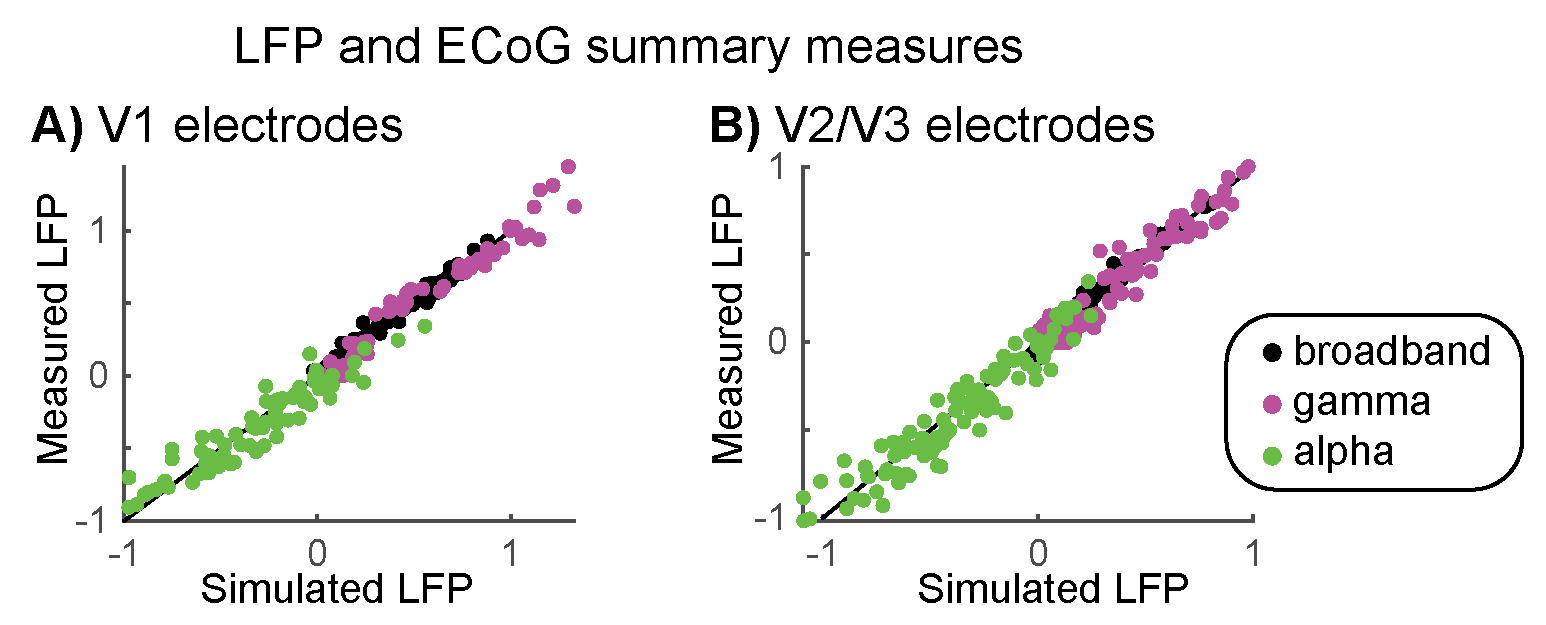

Supplement: S9 Fig — Every dot represents the broadband (black), gamma (magenta), or alpha (green) power change for one electrode, one stimulus condition. The power changes in the LFP are driven by changes in parameters C1, C2, and C3. We fitted these parameters such that the simulated LFP values for broadband, gamma, and alpha nicely match the measured values. (TIF) [file pbio.2001461.s009.tif]
